# Pro-inflammation NF-κB signaling triggers a positive feedback via enhancing cholesterol accumulation in liver cancer cells

**DOI:** 10.1186/s13046-017-0490-8

**Published:** 2017-01-18

**Authors:** Mingyan He, Wenhui Zhang, Yinying Dong, Lishun Wang, Tingting Fang, Wenqing Tang, Bei Lv, Guanglang Chen, Biwei Yang, Peixin Huang, Jinglin Xia

**Affiliations:** 1 Liver Cancer Institute, Zhongshan Hospital, Fudan University, Shanghai, 200032 China; 2 Cancer Hospital of Baotou City, Inner Monggolia, 014030 China; 3 Minhang Hospital, Fudan University, Shanghai, 201100 China

**Keywords:** Lipopolysaccharide, Nuclear factor-kappa B, Cholesterol accumulation, microRNA-195, Hepatocellular carcinoma

## Abstract

**Background:**

Hepatocellular carcinoma (HCC) develops in a complex microenvironment characterized by chronic inflammation. In recent years, cholesterol metabolic abnormalities have been implicated the importance in cancer cell physiology. This study was designed to investigate the relationship between inflammation and cholesterol accumulation in HCC cells.

**Methods:**

Human HCC cells HepG2 and Huh7 were cultured and stimulated with lipopolysaccharide (LPS) for 24 h. The changes of HCC cells related to cholesterol metabolism including intracellular cholesterol concentrations, cholesterol uptake, and the expression of cholesterol-related genes 3-hydroxy-3-methylglutaryl-CoA reductase (HMGCR), LDL receptor (LDLR), sterol regulatory element-binding transcription factor 2 (SREBF2), and proprotein convertase subtilisin/kexin 9 (PCSK9) were comparatively analyzed. Simultaneously, the effects of nuclear factor-kappa B (NF-κB) signaling pathway on cholesterol metabolism were clarified by knocking-down of nuclear factor kappa-B kinase subunit alpha (IKKα) and TGF-beta-activated kinase 1 and MAP3K7-binding protein 3 (TAB3) via RNAi and microRNA (miR)-195. Subsequently, the roles of cholesterol accumulation in LPS induced pro-inflammatory effects were further investigated.

**Results:**

Pro-inflammatory factor LPS significantly increased intracellular cholesterol accumulation by upregulating the expression of HMGCR, LDLR, and SREBF2, while downregulating the expression of PCSK9. These effects were revealed to depend on NF-κB signaling pathway by knocking-down and overexpression of IKKα and TAB3. Additionally, miR-195, a regulator directly targeting IKKα and TAB3, blocked the effects of cholesterol accumulation, further supporting the critical role of pro-inflammation NF-κB signaling in regulating cholesterol accumulation. Intriguingly, the accumulation of cholesterol conversely exerted an augmented pro-inflammation effects by further activating NF-κB signaling pathway.

**Conclusions:**

These results indicated that pro-inflammation effects of NF-κB signaling could be augmented by a positive feedback via enhancing the cholesterol accumulation in liver cancer cells.

## Background

Metabolic reprogramming in the uncontrolled proliferation of cancer cells has been thought to play important roles in cancer biology [1]. Recently, many studies have demonstrated that cholesterol accumulate in a series of human cancers, including breast [2], colon [3], prostate [4], HCC [5], and others [6]. In addition, enzymes involved in cholesterol metabolism have been reported abnormal expression in cancer tissues [7, 8]. For instance, cholesterol acyltransferase (ACAT)2, is found to be induced and promotes esterification of excess oxysterols for secretion to avoid cytotoxicity in a subset of hepatocellular carcinomas (HCCs) for tumor growth [9], suggestive of a specific cholesterol metabolic pathway in HCCs. On the one hand, cholesterol is needed for the synthesis of membranes, signaling molecules, lipid raft formation, and other factors to support the rapid growth of tumor cells [8]. On the other hand, cholesterol oxidized products, namely oxysterols, exhibit inhibitory activities in cell growth and promote cell apoptosis and dampen antitumor responses [10–12]. However, mechanism and pathological significance underlying the aberrant cholesterol metabolism are still elusive.

Epidemiological studies have shown that 80% of HCCs develop in fibrotic or cirrhotic livers as a consequence of chronic liver injury [13, 14]. The NF-κB pathway, the key link of inflammatory responses, plays an important role in HCC promotion by increasing proliferation and preventing apoptosis [15]. In the inactive state, NF-κB transcription factors are complexed in the cytoplasm, either with members of the inhibitor of κB (IκB) family (in the canonical pathway) or with the NF-κB precursor p100 (in the non-canonical pathway). Lipopolysaccharide (LPS), a component of the gram-negative bacterial wall, could activate NF-κB pathway and stimulate inflammatory responses. Previous studies have demonstrated that LPS/NF-κB signaling pathway promotes atherosclerotic progression and macrophage foam cell formation by increasing intracellular cholesterol accumulation [16, 17]. These reports inspired us to hypothesize whether there is a crosstalk between NF-κB signaling and cholesterol metabolism in cancer cells.

In the present study, we investigated the relationship between inflammation and cholesterol metabolism, and found that pro-inflammatory factor Lipopolysaccharide (LPS) increased intracellular cholesterol accumulation through NF-κB pathway in HCC cells. Cholesterol accumulation conversely promoted LPS/NF-κB pathway and inflammatory responses.

## Methods

### Cell culture

Human HCC cells HepG2 and Huh7 were purchased from Cell Bank of the institute of Biochemistry and Cell Biology, China Academy of Sciences, Shanghai, China. Cells were cultured in Dulbecco’s Modified Eagle’s Medium (DMEM) supplemented with 10% (v/v) fetal bovine serum (Gibco, BRL, USA), 10ug/mL streptomycin sulfate and 100ug/mL penicillin G. All cells were incubated at 37 °C in a humidified atmosphere containing 5% CO2.

### LDL preparation

LDL was purchased from AngYu Biotechnologies (Shanghai, China). It is isolated from blood bank produced human plasma. It is purified via ultracentrifugation to homogeneity determined by agarose gel electrophoresis. Each lot is analyzed on agarose gel electrophoresis for migration versus LDL.

### Measurement of intracellular cholesterol

Total cellular cholesterol measurement was determined with a detection kit from Applygen (Peking, China) according to the manufacturer’s protocol. Intracellular lipids were extracted using a chloroform-methanol (2:1) mix and dried under vacuum. Total cholesterol contents were measured by an enzymatic assay. The value was normalized against total protein concentration from each sample, as determined with Bicinchoninic Acid assay (Beyotime biotechnology, China).

### DIL-LDL uptake

The low density lipoprotein labeled with 1,1′-Dioctadecyl-3,3,3′,3′-tetramethyl- indocarbocyanideperchlorate (DIL-LDL) (Biomedical Technologies Inc., MA, USA) uptake represented the ability of cell cholesterol uptake. HCC cells were treated with different addressment, then changed to serum-free medium and incubated with 10ug/ml DIL-LDL at 37 °C for 5 h. The cells were detached by trypsin, washed and suspended by PBS and analyzed by flow cytometry using FL2 emission filter (FACScan, BD Biosciences, San Jose, CA, USA). Data were analyzed with FlowJo software.

### Total RNA isolation and quantitative real-time quantitative PCR

Total RNA, containing miRNA, was isolated from HCC cells with TRIzol reagent (Invitrogen, CA, USA) according to the provided protocol. For mRNA analysis, complementary DNA was synthesized using reverse transcription reagent kit (TaKaRa, Japan) from RNA of 500 ng. Primers for PCR were shown in Table 1. Quantitative PCR amplification was performed by ABI7500 real-time fluorescent measurement system (Applied Biosystems, USA) using PCR amplification Kit (TaKaRa, Japan). Values are derived from at least 3 separate experiments performed in duplicate and normalized to β-actin expression. The 2^-△△Ct^ method was used for the data analysis [18]. For miRNA analysis, the first-strand cDNA was synthesized using the reverse transcriptase with miRNAs-specific stem-loop primer (RiboBio, Guangzhou, China). PCR amplification was conducted in ABI7500 real-time fluorescent measurement system (Applied Biosystems, USA) using the Bulge-Loop miRNA qRT-PCR Starter Kit (RiboBio, Guangzhou, China) according to the protocol provided. The relative expression level of miRNA was normalized to that of endogenous control U6 by using 2^-△△Ct^ method.

**Table 1:** Primers for PCR used in this study

Gene Name	Forward primer (5′ → 3′)	Reverse primer (5′ → 3′)
PCSK9	AGACCCACCTCTCGCAGTC	GGAGTCCTCCTCGATGTAGTC
LDLR	GCTACCCCTCGAGACAGATG	CACTGTCCGAAGCCTGTTCT
SREBF2	GGTTGTCGGGTGTCATGGG	TTGCAGCATCTCGTCGATGT
HMGCR	GAGCGTGCGTAAGGTGAGG	ACAGAATCCTTGGATCCTCCAG
IKKα	GGCTTCGGGAACGTCTGTC	TTTGGTACTTAGCTCTAGGCGA
TAB3	AGCAGCCCACAGCTTGATATT	ACTAGGAGAATGGATACCCAGGT
MiR-195	GTCATTCCCTATTTCTTCTGCC	ATTGGACCATCTTCCCTCTGT
VCAM1	GGGAAGATGGTCGTGATCCTT	TCTGGGGTGGTCTCGATTTTA
MMP9	GGAGGTTCGACGTGAAGG	GGAACATCCGGTCCACCT
IL8	AGACAGCAGAGCACACAAGC	ATGGTTCCTTCCGGTGGT
TNFα	CTCGAACCCCGAGTGACAA	GCTGCCCCTCAGCTTGAG
IκBα	CATTGACATCAGCACCCAAG	CCACTCCATCCTGAAGGCTA
P100	CTCCTCTAGGCCCATGTCAG	AGCCTGGTAGACACGTACCG
β-actin	CGTGGACATCCGTAAAGACC	ACATCTGCTGGAAGGTGGAC

### Western blot

HCC cells were lysed in cell lysis buffer containing 1nM PMSF for 30 min at 4 °C. Lysates were collected by centrifugation at 12,000 rpm for 30 min at 4 °C. Proteins from cell lysates were separated on the SDS-PAGE and transferred onto PVDF membrane (Immobion-P Transfer Membrane, Millipore Corp., Billerica, MA, USA). The membrane was blocked with TBST containing 5% non-fat dry milk for 1 h and further incubated overnight at 4 °C with primary antibodies against PCSK9, LDLR, HMGCR, SREBF2 (Abcam, Cambridge, MA, USA), NF-κB p65, phospho-NF-κB p65, IKKα, TAB3 (Cell Signaling Technology, USA) and β-actin (Santa Cruz, CA, USA). After that, the membrane was incubated with horseradish peroxidase (HRP)-linked secondary antibodies (Santa Cruz Biotechnology, USA) for 2 h at room temperature. All protein bands were visualized using an electrochemiluminescence kit (Thermo, USA). Intensity of each protein band was quantified by Quantity One 4.6.2 software (Bio Rad).

### MiRNA inhibitor or mimic transfection

For transfections in 12-well plates, the HCC cells were seeded at a density of 1 × 10^5^ cells/well and incubated overnight and then transfected with 50 nM miR-195 mimic or 100 nM miR-195 inhibitor (GenePharma, Shanghai, China) using lipo2000 Transfection Agent (Invitrogen, USA) according to the manufacturer’s instructions. The corresponding negative sequence of mimic or inhibitors (GenePharma, Shanghai, China) were transfected with the same concentration as controls. At 24 h after the transfection, cells were harvested or further incubated with LPS (1000 ng/mL) for the following experiments.

### RNA interference

Gene silencing was performed by infecting HCC cells with siRNA oligonucleotides (GenePharma, Shanghai, China). The siRNA sequences were shown in Table 2. For transfections in 12-well plates, 1.0 × 10^5^ cells were seeded per well and incubated overnight, then transfected with 80nM TAB3-siRNA, IKKα-siRNA or negative sequence using lipo2000 Transfection Agent (Invitrogen, USA) according to the manufacturer’s protocol. At 24 h after the transfection, cells were harvested or further incubated with LPS (1000 ng/mL) for the following experiments.

**Table 2:** The sequences of siRNA used in this stud**y**

Identifier	Sense Primer Sequence (5′ → 3′)	Anti-sense Primer Sequence (3′ → 5′)
NC siRNA	CCACCUCUGAUCGAUUUAUdTdT	dTdTAUAAAUCGAUCAGAGGUGG
IKKα siRNA	GCAGGCUCUUUCAGGGACAdTdT	dTdTCGUCCGAGAAAGUCCCUGU
TAB3 siRNA	CCAAAGGUUCCAUGAAGAAdTdT	dTdTGGUUUCCAAGGUACUUCUU

### ELISA

HCC cells were stimulated with LPS (1000 ng/ml) for 24 h in serum-free medium in the presence or absence of LDL (200ug/ml). The concentrations of IL-8 and TNF-α in culture supernatants were measured with ELISA kits (R&D Systems, USA) according to the manufacturer’s instruction. Absorbance was recorded at 450 nm using a microplate reader.

### Statistical analysis

Data analysis was performed using SPSS16.0 statistical software (SPSS Inc, Chicago, IL). Student’s *t*-tests were used to assess significant differences among study groups. *P* < 0.05 was considered statistically significant. All experiments have been performed at least three times.

## Results

### Role of NF-κB signaling in cholesterol accumulation

**Fig. 1:**
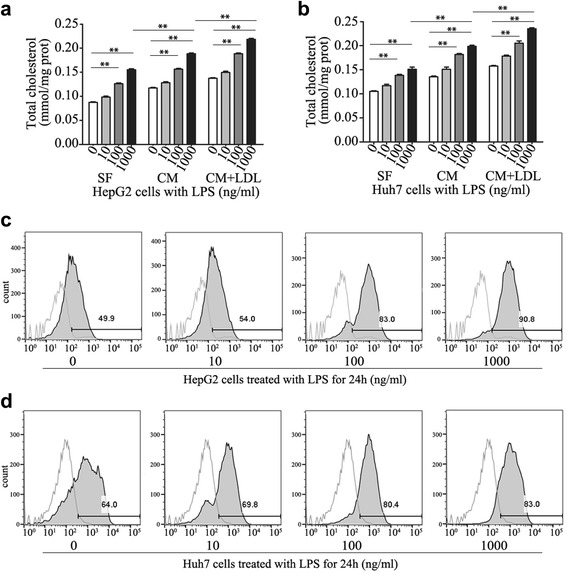
Effects of LPS on cholesterol metabolism in HCC cells. **a**, **b** Cells treated with 0, 10, 100, 1000 ng/ml LPS in serum free (SF) medium or complete medium (CM) in the absence or presence of LDL (200 ug/ml) for 24 h, the total cholesterol ester concentration was measured; **c**, **d** Cells treated with 0, 10, 100, 1000 ng/ml LPS for 24 h. DIL-LDL uptake was detected. Data represent the mean ± SD of triplicate samples; **P < 0.01

To assess whether cholesterol metabolism could be affected by NF-κB pathway in HCC cells, we monitored intracellular cholesterol levels in response to LPS at dosages ranging from 0 to 1000 ng/ml in serum free (SF) medium or complete medium (CM) or CM with LDL loading (200 μg/ml). As shown in Fig. 1a and b, LPS significantly increased the intracellular cholesterol concentration in a dose-dependent manner in HepG2 and Huh7 HCC cells. And the effects were not affected by constitutive cholesterol levels. Furthermore, we found that uptake of 3,3′-dioctadecylindocarbocyanine-low density lipoprotein (DIL-LDL) in HCC cells was stimulated by LPS, suggesting that it may increase native LDL cholesterol uptake via LDLR (Fig. 1c and d).

**Fig. 2:**
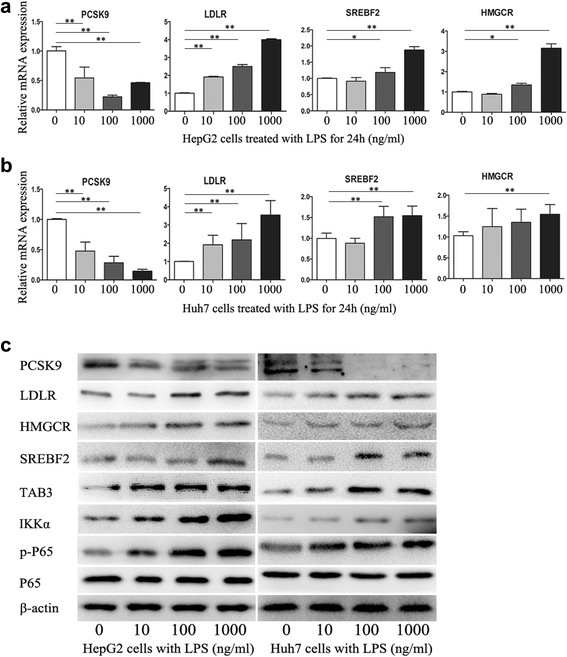
Expression of PCSK9, LDLR, HMGCR and SREBF2 in HCC cells stimulated with LPS. Cells treated with 0, 10, 100, 1000 ng/ml LPS for 24 h. The expression of PCSK9, LDLR, HMGCR, SREBF2 and the phosphorylation status of P65 were detected by qRT-PCR (**a**, **b**) and western blot (**c**). Data represent the mean ± SD of triplicate samples; *P < 0.05, **P < 0.01

Next, we examined the effect of LPS on the expression of proprotein convertase subtilisin/kexin 9 (PCSK9), LDLR, HMGCR and sterol regulatory element-binding transcription factor 2 (SREBF2) in HCC cells (Fig. 2a-c). After treatment with various concentrations of LPS for 24 h, PCSK9 mRNA and protein levels in HCC cells were significantly downregulated, and the expression of LDLR, HMGCR and SREBF2 were upregulated in a dose-dependent manner. In addition, LPS promotes the expression of IKKα, TAB3 and phosphorylated P65 (Fig. 2c), demonstrating the role of NF-κB pathway in HCC intracellular cholesterol accumulation.

### IKKα and TAB3 regulates LPS-induced cholesterol accumulation

**Fig. 3:**
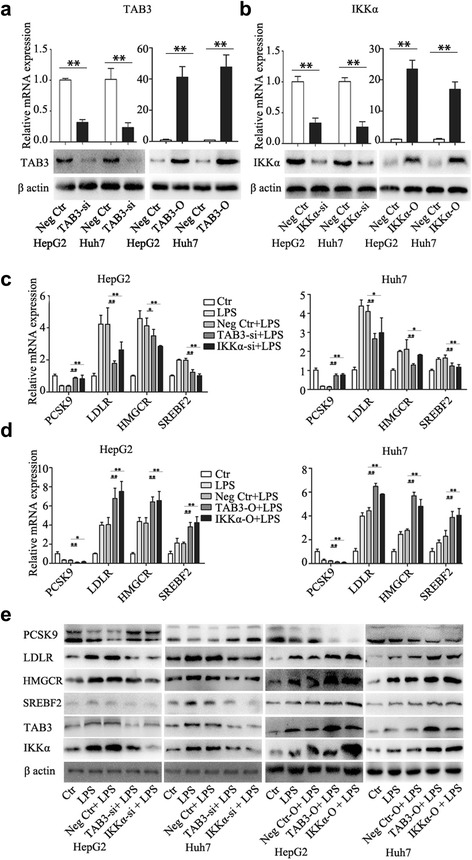
IKKα and TAB3 regulates cholesterol-related gene expression induced by LPS. **a**, **b** qRT-PCR and western blotting confirm the down- and up-regulation of IKKα or TAB3 expression in HCC cells. **c**, **d**, **e** Twenty-four hours after transfection, HCC cells were treated with LPS (1000 ng/ml) for 24 h, and then PCSK9, LDLR, HMGCR and SREBF2 expression were detected. Data represent the mean ± SD of triplicate samples; *P < 0.05, **P < 0.01

**Fig. 4:**
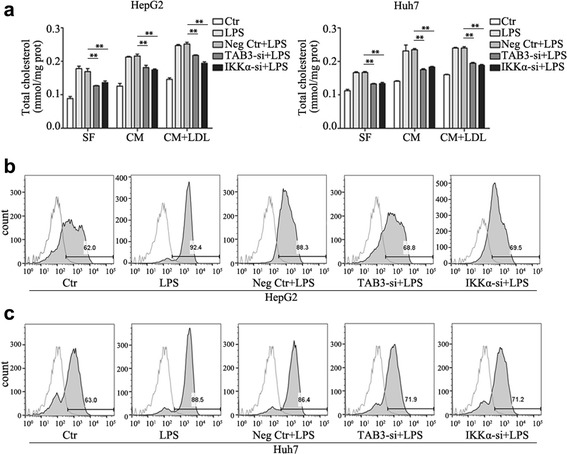
Down-regulation of IKKα and TAB3 expression suppressed LPS-induced cholesterol accumulation. **a** After transfection with IKKα, TAB3 siRNA or negative control for 24 h, cells treated with 1000 ng/ml LPS in serum free medium or complete medium in the absence or presence of LDL (200 ug/ml) for 24 h, then the total cholesterol ester concentration was measured. **b**, **c** After transfection with IKKα, TAB3 siRNA or negative control for 24 h, cells were exposed to LPS (1000 ng/mL) for 24 h, then DIL-LDL uptake was detected. Data represent the mean ± SD of triplicate samples; **P < 0.01

To further examine whether LPS mediated cholesterol accumulation by NF-κB pathway, we synthesized siRNAs specifically against nuclear factor kappa-B kinase subunit alpha (IKKα) and TGF-beta-activated kinase 1 and MAP3K7-binding protein 3 (TAB3) and overexpressed IKKα and TAB3 in HepG2 and Huh7 cells. The down- and up-regulated efficacy of TAB3 and IKKα was confirmed by quantitative real-time PCR and western blotting (Fig. 3a and b). Twenty-four hours after transfection, HCC cells were treated with LPS (1000 ng/ml) for 24 h, and PCSK9, LDLR, HMGCR, and SREBF2 expression, total cellular cholesterol levels, and uptake of DIL-LDL were measured at the endpoints. As expected, down-regulation of TAB3 and IKKα increased PCSK9 expression and decreased LDLR, HMGCR, and SREBF2 expression, while up-regulation of TAB3 and IKKα decreased PCSK9 expression and increased LDLR, HMGCR, and SREBF2 expression (Fig. 3c-e). Additionally, down-regulation of TAB3 and IKKα abrogated the effects of LPS on cholesterol accumulation (Fig. 4a–c). These findings indicated that TAB3 and IKKα were indeed functional in LPS-induced cholesterol accumulation.

### MiR-195 regulates cholesterol accumulation and cholesterol-related gene expression induced by LPS

**Fig. 5:**
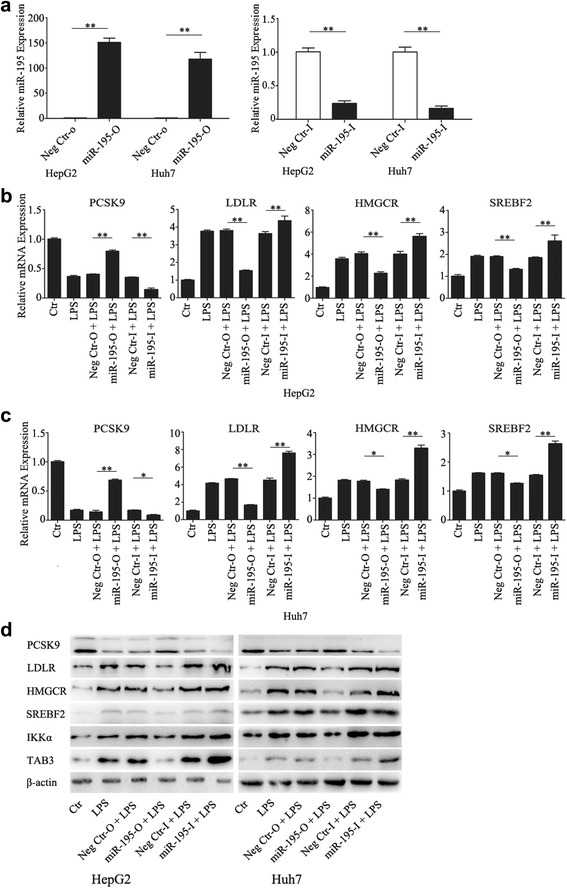
MiR-195 regulated cholesterol-related gene expression induced by LPS. **a** validation of miR-195 mimic and inhibitor by qRT-PCR at 24 h after transfection (**b**-**d**) After transfection with miR-195 mimic or inhibitor for 24 h, cells were exposed to LPS (1000 ng/mL) for 24 h. The expression levels of PCSK9, LDLR, HMGCR and SREBF2 in HCC cells were detected by qRT-PCR (**b**, **c**) and western blot (**d**). Data represent the mean ± SD of triplicate samples; *P < 0.05, **P < 0.01

**Fig. 6:**
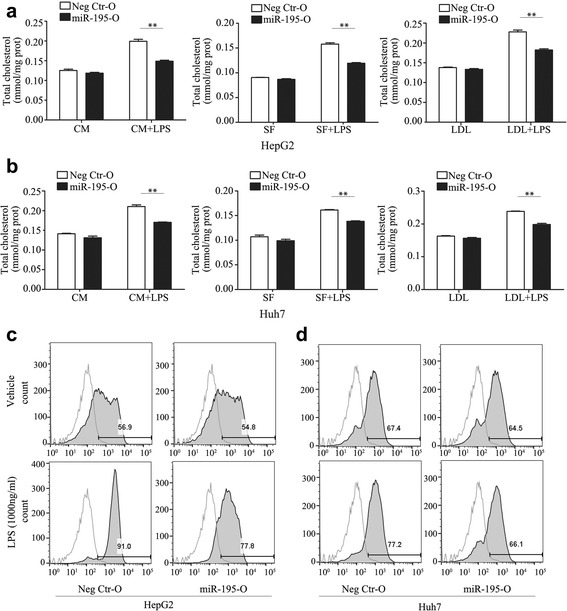
MiR-195 inhibited LPS-mediated cholesterol accumulation. **a**, **b** After transfection with miR-195 mimic for 24 h, cells treated with 1000 ng/ml LPS in serum free medium or complete medium in the absence or presence of LDL (200 ug/ml) for 24 h, then the total cholesterol ester concentration was measured. **c**, **d** After transfection with miR-195 mimic for 24 h, cells were exposed to LPS (1000 ng/mL) for 24 h. DIL-LDL uptake was detected. Data represent the mean ± SD of triplicate samples; **P < 0.01

Previous findings have shown that miR-195 decreases the activity of NF-κB signaling and the expression of NF-κB downstream effectors by directly targeting TAB3 and IKKα [19]. To further investigate the involvement of NF-κB signaling in cholesterol metabolism, the cells overexpressing or knocking down miR-195 were established by transfecting with miR-195 mimic, mir-195 inhibitor. As shown in Fig. 5a, these cells achieved more than a 100-fold upregulation and 80% downregulation of mRNA expression, respectively (Fig. 5a). Twenty-four hours after transfection with miR-195 mimic, miR-195 inhibitor, or negative control, HCC cells were treated with 1000 ng/mL LPS for another 24 h and then analyzed for intracellular cholesterol levels and related gene expression. Relative to the controls, mRNA and protein expression levels of PCSK9 were downregulated by LPS, and LDLR, HMGCR, and SREBF2 expression was upregulated. Overexpression of miR-195 increased PCSK9 expression and decreased LDLR, HMGCR, and SREBF2 expression, while knockdown of miR-195 decreased PCSK9 expression and increased LDLR, HMGCR, and SREBF2 expression (Figs. 5b–d). In addition, overexpression of miR-195 inhibited the total intracellular cholesterol level (Fig. 6a and b) and DIL-LDL uptake (Fig. 6c). These data indicate that miR-195 negatively regulates LPS-induced cholesterol accumulation and expression of cholesterol-related genes.

**Fig. 7:**
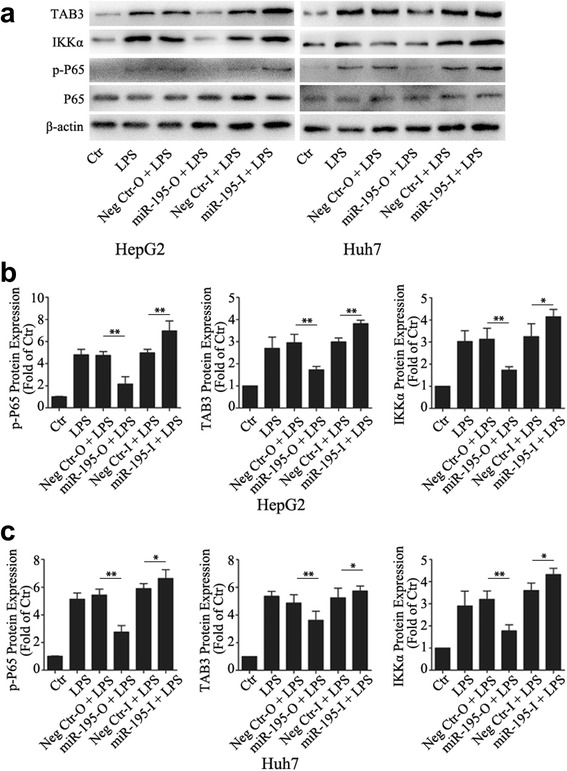
MiR-195 inhibited the activation of NF-κB pathway. **a**, **b**, **c** After transfection with miR-195 mimic for 24 h, cells treated with 1000 ng/ml LPS for 24 h, then the phosphorylation status of P65 and the expression levels of TAB3 and IKKα were detected by western blot. Data represent the mean ± SD of triplicate samples; *P < 0.05, **P < 0.01

Next, we investigated the phosphorylation status of p65 and the expression levels of TAB3 and IKKα, which were the direct gene targets of miR-195. We observed that overexpression of miR-195 significantly inhibited the phosphorylation status of p65 and the expression levels of TAB3 and IKKα, while knockdown of miR-195 increased the phosphorylation status of p65 and the expression levels of TAB3 and IKKα (Fig. 7), supporting our findings that NF-κB pathway played an important role in HCC intracellular cholesterol accumulation.

### Cholesterol increases LPS induced pro-inflammatory effects

**Fig. 8:**
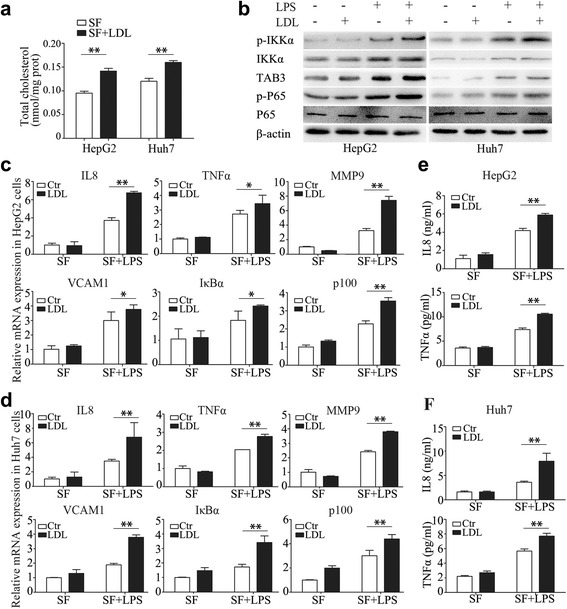
LDL increases LPS induced pro-inflammatory effects. LDL increases intracellular cholesterol contents (**a**), LDL significantly activated the phosphorylation of p65 and IKKα (**b**), increased the expression of NF-κB target genes (**c**, **d**), promoted the production of IL-8 and TNF-α in HCC cells with administration of LPS for 24 h (**e**, **f**). Data represent the mean ± SD of triplicate samples; *P < 0.05, **P < 0.01

LDL, containing 49% cholesterol, could increases intracellular cholesterol contents in HCC cells (Fig. 8a). To elucidate the influence of cholesterol accumulation in inflammatory responses, we investigated the phosphorylation status of NF-κB signaling pathway and the expression levels of 20 NF-κB downstream effectors in the absence or presence of LDL loading. LPS at 1000 ng/ml activated NF-κB signaling pathway and stimulated the expression of NF-κB downstream effectors, while the phosphorylation level of p65 and IKKα (Fig. 8b) and the mRNA abundance of six effectors (Fig. 8c and d) and cytokines production (Fig. 8e and f) was further increased by LDL at 200ug/ml in both HepG2 and Huh7 cells. The data suggest that accumulated cholesterol increases LPS/NF-κB-induced inflammatory effects by increasing p-IKKα in HCC cells.

## Discussion

**Fig. 9:**
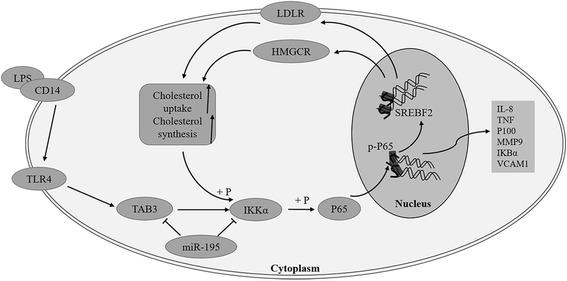
Illustration of the schematic representation of the working

HCC, among the most malignant of human cancers [20], develops in a complex microenvironment characterized by chronic inflammation [13, 14]. Emerging evidence has demonstrated that perpetuating hepatic inflammation promotes HCC initiation and progression [13, 21, 22]. Meanwhile, cholesterol metabolic abnormalities have been implicated the importance in cancer cell physiology in recent years [2–4, 23]. However, the relationship between the aberrant cholesterol metabolism and inflammation in HCCs are not completely understood. In the present study, we demonstrated that LPS/NF-κB pathway induced a significant increase in the intracellular cholesterol concentration and in DIL-LDL uptake in HCC cells, suggesting a key role of NF-κB pathway in promoting cholesterol accumulation. Additionally, the cholesterol accumulation conversely promoted LPS/NF-κB-induced inflammatory effects, indicating a positive feedback of inflammation and cholesterol metabolism (Fig. 9).

MicroRNAs (miRNAs) are a class of small non-coding RNAs, which repress translation or induce cleavage of target mRNAs by base pairing with their 3′ untranslated regions. MiR-195 is a member of the miR-15/16/195/424/497 family [24]. It has been reported that miR-195 suppresses cancer development and is downregulated in multiple types of cancer, such as prostate [25, 26], lung [27], osteosarcoma [28], HCCs [29], and so forth. Genome-wide screening suggests that miR-195 mediates NF-κB activity by directly targeting the expression of IKKα TAB3 in HCCs [19]. Here, we demonstrated that not only miR-195 but also its targeted genes TAB3 and IKKα mediate LPS/NF-κB-induced cholesterol accumulation and cholesterol-related gene expression, providing further evidence that NF-κB pathway play an important role in HCC intracellular cholesterol accumulation.

PCSK9, the ninth member of the proteinase K subfamily of subtilases, plays an important role in post-transcriptional degradation of LDLR, which decreases intracellular cholesterol uptake [30–32]. Decreased PCSK9 and increased LDLR expression have been demonstrated in HCC tissues, supporting a constant cholesterol supply in the HCC microenvironment [33]. HMGCR is the rate-limiting enzyme in *de novo* synthesis of cholesterol in vivo. Recent studies have reported that HMGCR is upregulated in several types of cancer including gastric [34], ovarian [7] and breast cancers [35], suggesting that HMGCR plays an oncogenic role. SREBF2 is a membrane-bound transcription factor that regulates cholesterol homeostasis in cells. It has been demonstrated that PCSK9, LDLR, and HMGCR expression are co-regulated by SREBF2 [36–38]. When cholesterol levels fall, SREBF2 is activated to up-regulate the expression of genes responsible for cholesterol synthesis, such as HMGCR, and for cholesterol uptake, such as LDLR. In this study, the expression of PCSK9, LDLR, HMGCR, and SREBF2 were investigated in HCC cells after stimulation with LPS. We found that LPS significantly inhibited the expression of PCSK9 and increased LDLR, HMGCR, and SREBF2 expression, suggesting that LPS may increase native LDL cholesterol uptake via LDLR and promote *de novo* cholesterol synthesis via HMGCR.

There are growing evidences that cholesterol, as an important molecule, impacts upon cancer cell physiology, however, the concrete role of cholesterol in cancer progression remains elusive and controversial. Analyses of the cancer Genome Atlas (TCGA) database revealed a correlation between increased activity of the cholesterol synthesis pathway and decreased survival in patients with sarcoma, acute myeloid leukemia and melanoma [39, 40], supporting the concept that cholesterol promotes carcinogenesis. However, some epidemiological studies have reported objective observation that poor prognosis in HCC patients were linked to decreased serum cholesterol [41, 42]. In this study, we have suggested that cholesterol further activated the NF-κB signaling pathway and promotes the expression of NF-κB target genes, indicating the pro-inflammatory effects of cholesterol in HCC cells.

## Conclusions

In summary, we have experimentally demonstrated that LPS/NF-κB signaling pathway triggers an increase in intracellular cholesterol levels by promoting the expression of HMGCR and LDLR in HCC cells. Cholesterol accumulation conversely promotes LPS/NF-κB induced pro-inflammatory effects*.* MiR-195, as a regulator of NF-κB pathway, inhibited cholesterol accumulation by decreasing the expression of TAB3 and IKKα. These data provide us with a better understanding of the relationship between LPS/NF-κB pathway and cholesterol abnormalities in cancer cells.

## Abbreviations

DIL-LDL: Low density lipoprotein labeled with 1,1′-Dioctadecyl-3,3,3′,3′-tetramethyl-indocarbocyanideperchlorate
HCC: Hepatocellular carcinoma
HMGCR: 3-hydroxy-3-methylglutaryl-CoA reductase
IKKα: Nuclear factor kappa-B kinase subunit alpha
LDLR: LDL receptor
LPS: Lipopolysaccharide
miR-195: microRNA-195
NF-κB: Nuclear factor-kappa B
PCSK9: Proprotein convertase subtilisin/kexin 9
SREBF2: Sterol regulatory element-binding transcription factor 2
TAB3: TGF-beta-activated kinase 1 and MAP3K7-binding protein 3

## References

[1] Warburg O (1956). On the origin of cancer cells. Science.

[2] de Gonzalo-Calvo D, López-Vilaró L, Nasarre L, Perez-Olabarria M, Vázquez T, Escuin D, Badimon L, Barnadas A, Lerma E, Llorente-Cortés V (2015). Intratumor cholesteryl ester accumulation is associated with human breast cancer proliferation and aggressive potential: a molecular and clinicopathological study. BMC Cancer.

[3] Accioly MT, Pacheco P, Maya-Monteiro CM, Carrossini N, Robbs BK, Oliveira SS, Kaufmann C, Morgado-Diaz JA, Bozza PT, Viola JPB (2008). Lipid bodies are reservoirs of cyclooxygenase-2 and sites of prostaglandin-E2 synthesis in colon cancer cells. Cancer Res.

[4] Yue S, Li J, Lee S, Lee HJ, Shao T, Song B, Cheng L, Masterson TA, Liu X, Ratliff TL, Cheng J (2014). Cholesteryl ester accumulation induced by PTEN loss and PI3K/AKT activation underlies human prostate cancer aggressiveness. Cell Metab.

[5] Jiang J, Xu N, Zhang X, Wu C (2007). Lipids changes in liver cancer. J Zhejiang Univ Sci B.

[6] White C (1909). The occurence of crystals in tumours. J Pathol Bacteriol.

[7] Stine JE, Guo H, Sheng X, Han X, Schointuch MN, Gilliam TP, Gehrig PA, Zhou C, Bae-Jump VL (2016). The HMG-CoA reductase inhibitor, simvastatin, exhibits anti-metastatic and anti-tumorigenic effects in ovarian cancer. Oncotarget.

[8] Abramson HN (2011). The lipogenesis pathway as a cancer target. J Med Chem.

[9] Lu M, Hu XH, Li Q, Xiong Y, Hu GJ (2013). A specific cholesterol metabolic pathway is established in a subset of HCCs for tumor growth. J Mol Cell Biol.

[10] Gill S, Chow R, Brown AJ (2008). Sterol regulators of cholesterol homeostasis and beyond: the oxysterol hypothesis revisited and revised. Prog Lipid Res.

[11] Lanterna C, Musumeci A, Raccosta L, Corna G, Moresco M, Maggioni D, Fontana R, Doglioni C, Bordignon C, Traversari C, Russo V (2016). The administration of drugs inhibiting cholesterol/oxysterol synthesis is safe and increases the efficacy of immunotherapeutic regimens in tumor-bearing mice. Cancer Immunol Immunother.

[12] Traversari C, Russo V (2012). Control of the immune system by oxysterols and cancer development. Curr Opin Pharmacol.

[13] Luedde T, Schwabe RF (2011). NF-κB in the liver—linking injury, fibrosis and hepatocellular carcinoma. Nat Rev Gastro Hepat.

[14] Fattovich G, Stroffolini T, Zagni I, Donato F (2004). Hepatocellular carcinoma in cirrhosis: incidence and risk factors. Gastroenterology.

[15] Dapito DH, Mencin A, Gwak G, Pradere J, Jang M, Mederacke I, Caviglia JM, Khiabanian H, Adeyemi A, Bataller R, Lefkowitch JH, Bower M, Friedman R, Sartor RB, Rabadan R, Schwabe RF (2012). Promotion of hepatocellular carcinoma by the intestinal microbiota and TLR4. Cancer Cell.

[16] Li LC, Varghese Z, Moorhead JF, Lee CT, Chen JB, Ruan XZ (2013). Cross-talk between TLR4-MyD88-NF- B and SCAP-SREBP2 pathways mediates macrophage foam cell formation. Am J Physiol Heart Circ Physiol.

[17] Lehr HA, Sagban TA, Ihling C, Zahringer U, Hungerer KD, Blumrich M, Reifenberg K, Bhakdi S (2001). Immunopathogenesis of atherosclerosis: endotoxin accelerates atherosclerosis in rabbits on hypercholesterolemic diet. Circulation.

[18] Livak KJ, Schmittgen TD (2001). Analysis of relative gene expression data using real-time quantitative PCR and the 2(−delta delta C(T)) method. Methods.

[19] Ding J, Huang S, Wang Y, Tian Q, Zha R, Shi H, Wang Q, Ge C, Chen T, Zhao Y, Liang L, Li J, He X (2013). Genome-wide screening reveals that miR-195 targets the TNF-alpha/NF-kappaB pathway by down-regulating IkappaB kinase alpha and TAB3 in hepatocellular carcinoma. Hepatology.

[20] Venook AP, Papandreou C, Furuse J, de Guevara LL (2010). The incidence and epidemiology of hepatocellular carcinoma: a global and regional perspective. Oncologist.

[21] Wilson CL, Jurk D, Fullard N, Banks P, Page A, Luli S, Elsharkawy AM, Gieling RG, Chakraborty JB, Fox C, Richardson C, Callaghan K, Blair GE, Fox N, Lagnado A, Passos JF (2015). NFkappaB1 is a suppressor of neutrophil-driven hepatocellular carcinoma. Nat Commun.

[22] Sanz-Cameno P, Trapero-Marugan M, Chaparro M, Jones EA, Moreno-Otero R (2010). Angiogenesis: from chronic liver inflammation to hepatocellular carcinoma. J Oncol.

[23] Ribas V, García-Ruiz C, Fernández-Checa JC (2016). Mitochondria, cholesterol and cancer cell metabolism. Clin Transl Med.

[24] Griffiths-Jones S, Saini HK, van Dongen S, Enright AJ (2008). miRBase: tools for microRNA genomics. Nucleic Acids Res.

[25] Guo J, Wang M, Liu X (2015). MicroRNA-195 suppresses tumor cell proliferation and metastasis by directly targeting BCOX1 in prostate carcinoma. J Exp Clin Cancer Res.

[26] Cai C, Chen QB, Han ZD, Zhang YQ, He HC, Chen JH, Chen YR, Yang SB, Wu YD, Zeng YR, Qin GQ, Liang YX, Dai QS, Jiang FN, Wu SL, Zeng GH (2015). miR-195 inhibits tumor progression by targeting RPS6KB1 in human prostate cancer. Clin Cancer Res.

[27] Liu B, Qu J, Xu F, Guo Y, Wang Y, Yu H, Qian B (2015). MiR-195 suppresses non-small cell lung cancer by targeting CHEK1. Oncotarget.

[28] Han K, Chen X, Bian N, Ma B, Yang T, Cai C, Fan Q, Zhou Y, Zhao TB (2015). MicroRNA profiling identifies MiR-195 suppresses osteosarcoma cell metastasis by targeting CCND1. Oncotarget.

[29] Xu T, Zhu Y, Xiong Y, Ge YY, Yun JP, Zhuang SM (2009). MicroRNA-195 suppresses tumorigenicity and regulates G1/S transition of human hepatocellular carcinoma cells. Hepatology.

[30] Maxwell KN, Breslow JL (2004). Adenoviral-mediated expression of Pcsk9 in mice results in a low-density lipoprotein receptor knockout phenotype. Proc Natl Acad Sci U S A.

[31] Nassoury N, Blasiole DA, Tebon OA, Benjannet S, Hamelin J, Poupon V, McPherson PS, Attie AD, Prat A, Seidah NG (2007). The cellular trafficking of the secretory proprotein convertase PCSK9 and its dependence on the LDLR. Traffic.

[32] Lagace TA, Curtis DE, Garuti R, McNutt MC, Park SW, Prather HB, Anderson NN, Ho YK, Hammer RE, Horton JD (2006). Secreted PCSK9 decreases the number of LDL receptors in hepatocytes and in livers of parabiotic mice. J Clin Invest.

[33] Bhat M, Skill N, Marcus V, Deschenes M, Tan X, Bouteaud J, Negi S, Awan Z, Aikin R, Kwan J, Amre R, Tabaries S, Hassanain M, Seidah NG, Maluccio M, Siegel P (2015). Decreased PCSK9 expression in human hepatocellular carcinoma. BMC Gastroenterol.

[34] Chushi L, Wei W, Kangkang X, Yongzeng F, Ning X, Xiaolei C (2016). HMGCR is up-regulated in gastric cancer and promotes the growth and migration of the cancer cells. Gene.

[35] Singh R, Yadav V, Kumar S, Saini N (2015). MicroRNA-195 inhibits proliferation, invasion and metastasis in breast cancer cells by targeting FASN, HMGCR, ACACA and CYP27B1. Sci Rep.

[36] Jeong HJ, Lee HS, Kim KS, Kim YK, Yoon D, Park SW (2008). Sterol-dependent regulation of proprotein convertase subtilisin/kexin type 9 expression by sterol-regulatory element binding protein-2. J Lipid Res.

[37] Dubuc G, Chamberland A, Wassef H, Davignon J, Seidah NG, Bernier L, Prat A (2004). Statins upregulate PCSK9, the gene encoding the proprotein convertase neural apoptosis-regulated convertase-1 implicated in familial hypercholesterolemia. Arterioscler Thromb Vasc Biol.

[38] Horton JD, Goldstein JL, Brown MS (2002). SREBPs: activators of the complete program of cholesterol and fatty acid synthesis in the liver. J Clin Invest.

[39] Kuzu OF, Noory MA, Robertson GP (2016). The role of cholesterol in cancer. Cancer Res.

[40] Weinstein JN, Collisson EA, Mills GB, Shaw KR, Ozenberger BA, Ellrott K, Shmulevich I, Sander C, Stuart JM, Network CGAR (2013). The Cancer Genome Atlas Pan-Cancer analysis project. Nat Genet.

[41] Chen Z, Keech A, Collins R, Slavin B, Chen J, Campbell TC, Peto R (1993). Prolonged infection with hepatitis B virus and association between low blood cholesterol concentration and liver cancer. BMJ.

[42] Li WX (1993). Serum cholesterol and cancer mortality: eleven-year prospective cohort study on more than nine thousand persons. Zhonghua Liu Xing Bing Xue Za Zhi.

